# To the Editors of the Medical and Physical Journal

**Published:** 1804-11-01

**Authors:** 


### *To the Editors of the Medical and Physical Journal.*

Gentlemen,

THE investigation of circumstances the most interesting to humanity, of manifest importance to science, and grateful to the enquiring mind, although arduous the attempt and difficult the accomplishment, should be essayed with a philosophic composure and a strength of intellectual stability, incompatible with the faculties of derision or puerile frivolity.

What subject, at this critical epoch, can appear more important for the welfare of individuals, or solicits more the contemplation of humanity and the exertion of skill, than that object which has given rise to the zealous inquiry of the Old Campaigner? Whoever he is, my tribute of approbation not only for his efforts, which have as yet proved compleatly unsuccessful, but for his spirited and forcible address on the important occasion, in your Journal of August. With every expectation that the medical department of the army of Egypt, of which magna pars fui, will yet receive a faithful history of the death wound of Sir R. Abercrombie,

I am, &c.

*Edinburgh, September* 11, 1804. MEDICUS.

